# Dr. Robert Chambers

**Published:** 1911-09-16

**Authors:** 


The death is announced of Dr. Robert Chambers, of Belfast, on August 28. He was a well-known lover of flowers and a most successful rose-grower. Dr. Chambers studied both at Edinburgh and at Dublin, taking diplomas at those Universities in 1868 and 1870 respectively. He retired from active practice about two years ago, having till then resided in London, where he was Superintendent of the Marylebone Medical Mission, physician to the London Medical Mission and to the Church Army Training Homes. He took an active interest each year in the Keswick Convention.

